# Carbachol-Induced Reduction in the Activity of Adult Male Zebra Finch RA Projection Neurons

**DOI:** 10.1155/2016/7246827

**Published:** 2016-01-20

**Authors:** Wei Meng, Song-Hua Wang, Dong-Feng Li

**Affiliations:** ^1^School of Life Science, South China Normal University, Guangzhou, Guangdong 510631, China; ^2^Jiangxi Key Laboratory of Organic Chemistry, Jiangxi Science and Technology Normal University, Nanchang, Jiangxi 330013, China

## Abstract

Cholinergic mechanism is involved in motor behavior. In songbirds, the robust nucleus of the arcopallium (RA) is a song premotor nucleus in the pallium and receives cholinergic inputs from the basal forebrain. The activity of projection neurons in RA determines song motor behavior. Although many evidences suggest that cholinergic system is implicated in song production, the cholinergic modulation of RA is not clear until now. In the present study, the electrophysiological effects of carbachol, a nonselective cholinergic receptor agonist, were investigated on the RA projection neurons of adult male zebra finches through whole-cell patch-clamp techniques in vitro. Our results show that carbachol produced a significant decrease in the spontaneous and evoked action potential (AP) firing frequency of RA projection neurons, accompanying a hyperpolarization of the membrane potential, an increase in the evoked AP latency, afterhyperpolarization (AHP) peak amplitude, and AHP time to peak, and a decrease in the membrane input resistance, membrane time constant, and membrane capacitance. These results indicate that carbachol reduces the activity of RA projection neurons by hyperpolarizing the resting membrane potential and increasing the AHP and the membrane conductance, suggesting that the cholinergic modulation of RA may play an important role in song production.

## 1. Introduction

Songbirds have a unique learned behavior. Juvenile male songbirds learn their song from adult conspecific tutor songs during the sensitive period of song learning [1]. It has been demonstrated that motor production of song is controlled by the vocal motor pathway (VMP) in the pallium and brainstem [2]. The robust nucleus of the arcopallium (RA) is a premotor nucleus in the VMP. RA receives inputs from HVC (used as a proper name) of the VMP and also receives inputs from the lateral magnocellular nucleus of anterior nidopallium (LMAN) of the anterior forebrain pathway (AFP) that is necessary for song learning and adult song variability [3]. RA projection neurons project to brainstem respiratory and vocal control nuclei to form a part of song premotor pathway [4]. As well known, output of the AFP goes to both RA and HVC [5]. Considering the important function of the AFP on song learning, it was supposed that RA may play an important role in the regulation of song learning and song production.

Acetylcholine (ACh) is an ancient neurotransmitter. Cholinergic stimulation of the cerebral cortex is essential for tasks requiring attention [6]. The major cholinergic input to the mammalian cortex originates from the basal forebrain cholinergic system [7]. It was reported that the organization of the cholinergic systems in many regions of the avian brain (such as the basal forebrain) was quite similar to mammals [8]. Several studies showed that cholinergic fibers and neurons exist in several songbirds' song control nuclei including RA [9–11]. Then, the work of Li and Sakaguchi [12] indicated that the cholinergic fibers which innervate RA originate from the ventral paleostriatum (VP) in the basal forebrain of songbirds, and the VP is homologous to the nucleus basalis of Meynert of the basal forebrain cholinergic system of mammals. Some academics suggested that RA projection neurons are similar to pyramidal tract neurons of lower layer 5 of mammalian motor cortex [13, 14]. In mammals, cholinergic systems could potentially regulate motor cortex plasticity and learning [15]. This could suggest that the basal forebrain cholinergic system of songbirds might play an important role in learning and motor control of song.

A previous study found that, corresponding to an increase in ACh levels of RA, phosphoinositide turnover in synaptoneurosomes of RA increased during the most sensitive learning period, suggesting that ACh contributes to activities of RA neurons involved with song learning [16]. Although there is an evidence suggesting that nicotinic ACh receptors (nAChRs) are involved in the regulation of RA neuron activity [17], the cholinergic modulation of RA is not entirely clear until now. In order to better understand the cholinergic modulation of RA, we investigated the electrophysiological effects of carbachol, a nonselective cholinergic receptor agonist, on the RA projection neurons of adult male zebra finches using in vitro whole-cell current-clamp recording.

## 2. Material and Methods

### 2.1. Animals and Slice Preparation

All experiments were performed on adult male zebra finches (*Taeniopygia guttata*) (>120 days old), which were purchased from a local breeder, using an acute brain slice preparation in accordance with the university (scnu20070033) and national animal guidelines. The experimental methods had been described in our previous work [18]. In brief, birds were anesthetized with 10% chloral hydrate, and their brains were removed from the skull and immersed in ice cold, oxygenated (95% O_2_ and 5% CO_2_) slice solution consisting of (in mM) sucrose 248, KCl 5, NaHCO_3_ 28, glucose 10, MgSO_4_·7H_2_O 1.3, and NaH_2_PO_4_·H_2_O 1.26 (pH 7.4). Coronal brain slices (250 *μ*m thick) containing RA were obtained using a vibrating microtome (NVSLM1, World Precision Instruments, USA). Slices were collected in 37°C, oxygenated artificial cerebrospinal fluid (ACSF) consisting of (in mM) NaCl 125, NaHCO_3_ 25, NaH_2_PO_4_·H_2_O 1.27, KCl 2.5, MgSO_4_·7H_2_O 1.2, CaCl_2_ 2.0, and glucose 25 (pH 7.4). After 30 min, slices were allowed to recover at room temperature for at least 1 h prior to experiments.

### 2.2. Patch-Clamp Recording and Drug Application

After slices recovery, individual slices were transferred to a submerge-type recording chamber perfused with oxygenated ACSF (room temperature: 22–26°C) at the rate of 2.0 mL/min. RA neurons were visualized in the view of a BX51WI microscope connected with a DIC-IR video camera (Olympus, Japan) at high magnification (40x), and RA projection neurons were determined in terms of the distinct electrophysiological properties as described previously, time-dependent inward rectification induced by hyperpolarizing current, and regular firing induced by depolarizing current [19, 20]. All experiments were performed with conventional whole-cell patch recording under current-clamp configurations. The recording electrodes were fabricated from borosilicate glass pipettes (Sutter Instruments, USA) using a Flaming-Brown puller (P-97, Sutter Instruments, USA) and were filled with intracellular solution consisting of (in mM) KMeSO_4_ 120, NaCl 5, HEPES 10, EGTA 2, Mg-ATP 2, and Na_3_-GTP 0.3 (pH 7.3–7.4) with the electrode resistance 4–6 MΩ. RA projection neurons with resting membrane potential more positive than −55 mV or the series resistance (typically 10–20 MΩ) changes >30% were abandoned.

The effects of carbachol (Sigma-Aldrich, USA) on the intrinsic properties of RA projection neurons were tested by bath perfusion at the concentration of 0, 10, 20, 30, 40, and 50 *μ*M. Only one RA projection neuron was tested in a brain slice. Therefore, the sample number (*n*) in each experimental group represents the number of RA projection neurons recorded from different brain slices. Signals were amplified by a MultiClamp 700B (Axon Instruments, USA), low-pass-filtered at 5 kHz, and digitized by a Digidata 1440A (Axon Instruments, USA) at the sampling frequency of 10 kHz.

### 2.3. Data Analysis

The software pClamp 10.4 (Axon Instruments, USA) and Origin Pro 8.0 (Origin Lab, USA) were used for data acquisition and analysis. The time between the onset of the stimulus and the first evoked action potential (AP) was measured as the evoked AP latency. The membrane potential at the onset of an AP was taken as the AP threshold. The voltage difference between the peak of an AP and the AP threshold was measured as the peak amplitude. The halfway of AP amplitude was measured as the AP half-width. The voltage difference between the most negative voltage reached during the afterhyperpolarization (AHP) and the AP threshold was measured as the AHP peak amplitude. The AHP time to peak is the time of this minimum minus the time when the membrane potential crosses the AP threshold on descent from the AP peak [21]. A series of 600 ms hyperpolarizing current steps from −200 to 0 pA, step 20 pA with 10 s intervals, was applied to measure the membrane input resistance, calculated from the slope of the current-voltage curve. The membrane time constant was calculated by fitting a single exponential curve to the membrane potential change in response to −200 pA hyperpolarizing pulses. The membrane capacitance was calculated using the following equation: membrane capacitance = membrane time constant/membrane input resistance [22]. All data were expressed as means ± SEM and compared using paired two-tailed Student's *t*-test (*p* < 0.05 represents statistical significance).

## 3. Results

### 3.1. Effects of Carbachol on the Spontaneous AP Firing of RA Projection Neurons

**Figure 1:**
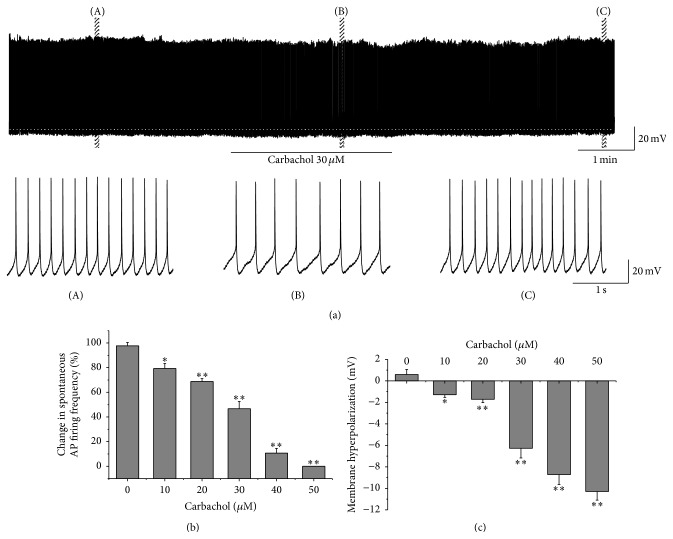
Carbachol produced a concentration-dependent decrease in the spontaneous AP firing frequency and induced a concentration-dependent membrane hyperpolarization in RA projection neurons. (a) A representative whole-cell recording showed the reversible effects of 30 *μ*M carbachol on the spontaneous AP firing frequency and membrane hyperpolarization. (A, B, C) Traces from the box area in (a) in expanded time scales. (b) Inhibitory effects of carbachol on the spontaneous AP firing frequency. The number of neurons was 6, 6, 7, 11, 7, and 7 in 0, 10, 20, 30, 40, and 50 *μ*M carbachol, respectively. (c) Hyperpolarization effects of carbachol on the resting membrane potential. The number of neurons was the same to (b). ^*∗*^ *p* < 0.05; ^*∗∗*^ *p* < 0.01, paired *t* test, as compared with 0 *μ*M carbachol group.

The spontaneous AP firing of RA projection neurons was recorded with the conventional whole-cell patch recording under current-clamp configurations at zero holding current (Figure 1(a)). We tested, respectively, the effects of 0, 10, 20, 30, 40, and 50 *μ*M carbachol on the spontaneous AP firing. As shown in Figure 1(b), a carbachol concentration-dependent decrease in the spontaneous AP firing frequency was observed. For instance, the application of 30 *μ*M carbachol significantly decreased the spontaneous AP firing frequency from 4.70 ± 0.56 to 2.18 ± 0.41 (Figure 1(b)). Moreover, the spontaneous AP firing was completely inhibited by 50 *μ*M carbachol (Figure 1(b)). Carbachol also induced a membrane hyperpolarization (Figures 1(a) and 1(c)). The effects of carbachol on RA projection neurons were reversible, because the spontaneous AP firing frequency and membrane potential gradually returned to the control level after carbachol washout (Figure 1(a)).

### 3.2. Effects of Carbachol on the Evoked AP Firing of RA Projection Neurons

In order to further determine the effects of carbachol on activity of RA projection neurons, we deliberately chose a moderate concentration (30 *μ*M; Figures 1(b) and 1(c)) of carbachol to test its effects on the evoked AP firing.

**Figure 2:**
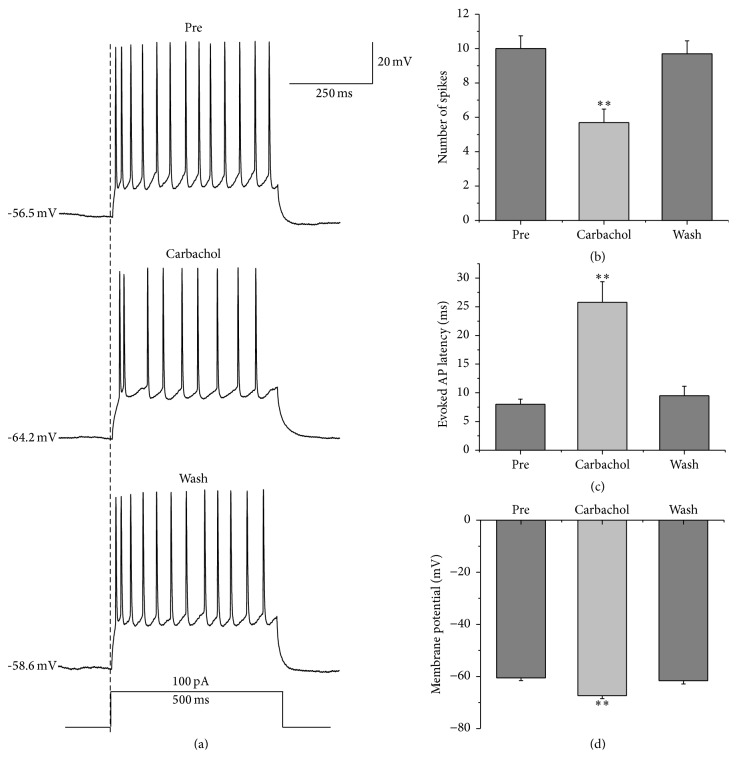
Effects of 30 *μ*M carbachol on the AP firing of RA projection neurons evoked by a depolarizing stimulus of 100 pA amplitude at 500 ms duration. (a) Sample traces showed that carbachol decreased the number of spikes evoked by depolarizing step pulse and induced membrane hyperpolarization. (b) The number of spikes evoked by depolarizing step pulse was significantly decreased in the presence of carbachol (*n* = 23). (c) The evoked AP latency was significantly increased in the presence of carbachol (*n* = 20, the latency is determined from the time between the onset of the stimulus and the first evoked AP for each cell). (d) Membrane potential was significantly hyperpolarized in the presence of carbachol (*n* = 23). *∗∗* means *p* < 0.01, paired *t* test.

Here we first evoked RA projection neurons' AP firing with a depolarizing stimulus of 100 pA amplitude at 500 ms duration and then tested the effects of carbachol on this evoked neuronal activity (Figure 2(a)). The results showed that the application of 30 *μ*M carbachol significantly decreased the number of spikes evoked by the depolarizing stimulus from 10.00 ± 0.74 to 5.70 ± 0.79, and the number of spikes returned to 9.70 ± 0.76 after carbachol washout (Figure 2(b)), indicating that the effects of carbachol are reversible. Moreover, 30 *μ*M carbachol markedly increased the evoked AP latency from 8.01 ± 0.90 ms to 25.78 ± 3.58 ms, and the evoked AP latency returned to 9.50 ± 1.65 ms after carbachol washout (Figure 2(c)), indicating that carbachol decelerated the firing of APs given the same stimulation strength [23]. Meanwhile, 30 *μ*M carbachol induced the membrane potential to hyperpolarize from −60.52 ± 1.06 mV to −67.40 ± 1.08 mV, and the membrane potential returned to −61.65 ± 1.23 mV after carbachol washout (Figure 2(d)), indicating that the decrease of RA projection neuron excitability caused by carbachol may be due to the membrane hyperpolarization.

**Figure 3:**
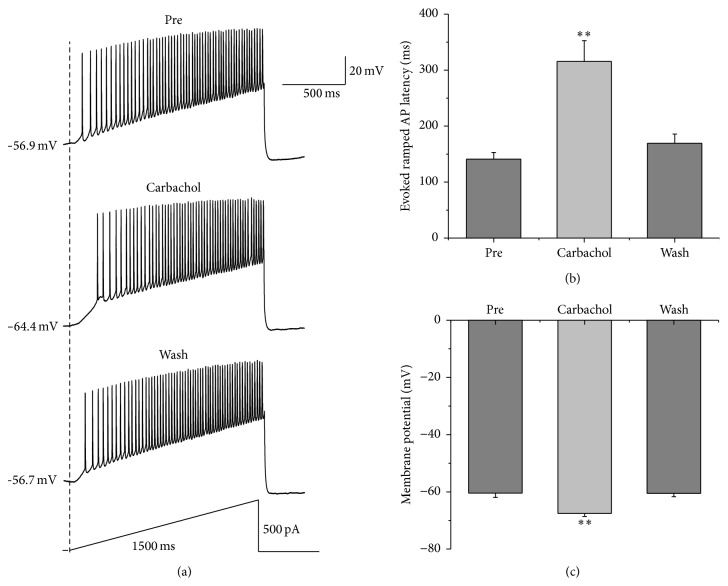
Effects of 30 *μ*M carbachol on the AP firing of RA projection neurons evoked by a depolarizing ramp stimulus. (a) Sample traces of AP firing evoked by a ramp current before, during, and after the application of carbachol. (b) The evoked ramped AP latency was significantly increased in the presence of carbachol (*n* = 23, the latency is determined from the time between the onset of the stimulus and the first evoked AP for each cell). (c) Membrane potential was significantly hyperpolarized in the presence of carbachol (*n* = 23). *∗∗* means *p* < 0.01, paired *t* test.

We further tested the reversible effects of carbachol on the evoked activity of RA projection neurons with another pattern of depolarizing stimulus, ramp, where intensity was increased from 0 to 500 pA in a linear manner within 1500 ms (Figure 3(a)). The results showed that the application of 30 *μ*M carbachol significantly increased the evoked ramped AP latency from 141.07 ± 11.80 ms to 315.56 ± 37.16 ms, and the evoked ramped AP latency returned to 169.37 ± 16.36 ms after carbachol washout (Figure 3(b)). An increase in the evoked ramped AP latency indicates that a stronger strength of the depolarizing stimulation was required to evoke AP firing in the presence of carbachol [23]. In addition, 30 *μ*M carbachol also induced the membrane potential to hyperpolarize from −60.44 ± 1.51 mV to −67.52 ± 1.09 mV, and the membrane potential returned to −60.52 ± 1.21 mV after carbachol washout (Figure 3(c)). These results further suggested that the excitability of RA projection neurons declined under the action of carbachol, which may be due to the membrane hyperpolarization.

### 3.3. Effects of Carbachol on the Other Intrinsic Properties of RA Projection Neurons

**Figure 4:**
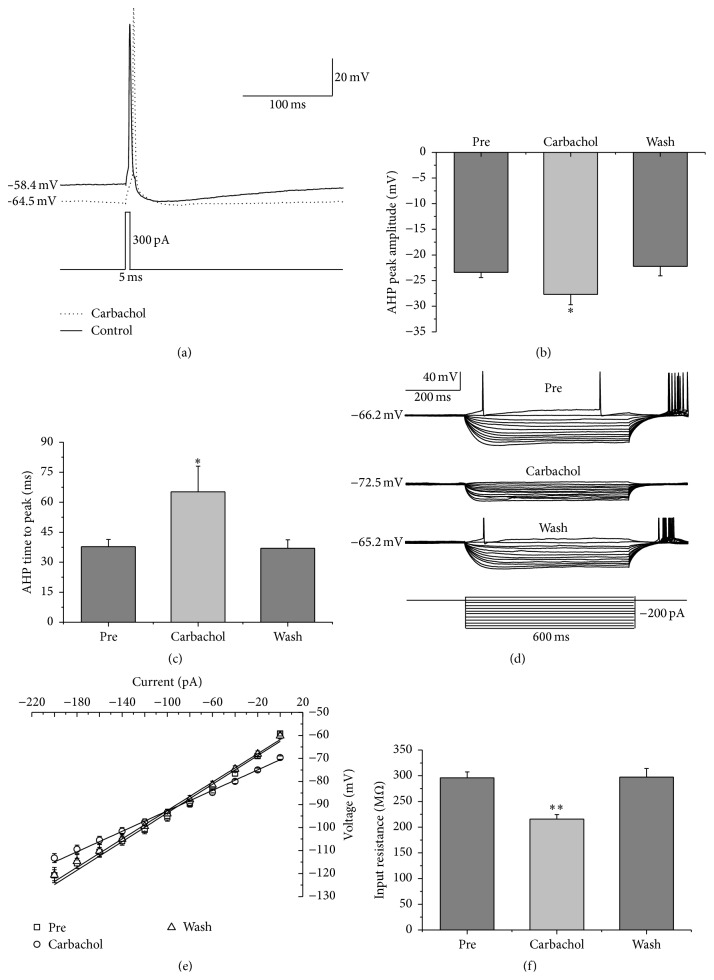
Effects of 30 *μ*M carbachol on the intrinsic properties of RA projection neurons. (a) Representative AP recordings in response to a depolarizing pulse before and during carbachol application. (b) AHP peak amplitude was significantly increased in the presence of carbachol (*n* = 19). (c) AHP time to peak was significantly increased in the presence of carbachol (*n* = 19). (d) Voltage responses of a neuron to a series of hyperpolarizing current steps before, during, and after carbachol application. (e) The current-voltage curves showed that there was a significant change in the slope by carbachol application (*n* = 34), indicating carbachol had an effect on membrane input resistance. (f) Membrane input resistance was significantly decreased in the presence of carbachol (*n* = 34). *∗* means *p* < 0.05, *∗∗* means *p* < 0.01, paired *t* test.

Finally, we analyzed the effects of carbachol on the intrinsic properties of the AP evoked by a depolarizing current pulse on RA projection neurons (Figure 4(a)). The results showed that, along with the membrane potential hyperpolarizing significantly and recovering gradually (Table 1), the AP's AHP peak amplitude and AHP time to peak were increased during 30 *μ*M carbachol application and recovered to the control level after carbachol washout (Table 1 and Figures 4(b) and 4(c)). But the AP threshold, peak amplitude, and half-width were unaffected (Table 1).

In addition, we also tested the effects of carbachol on the membrane input resistance, membrane time constant, and membrane capacitance of RA projection neurons. The results showed that the membrane input resistance of RA projection neurons, measured with a series of hyperpolarizing current steps (Figure 4(d)), was significantly decreased during 30 *μ*M carbachol application and recovered to the control level after carbachol washout (Table 1 and Figures 4(e) and 4(f)). The membrane time constant and membrane capacitance of RA projection neurons were significantly decreased as well during carbachol application and recovered gradually after carbachol washout (Table 1). These results indicated that the effects of carbachol on the intrinsic properties of RA projection neurons are reversible.

## 4. Discussion

In the present study, we have found that carbachol produced a hyperpolarization of membrane potential and an increase in the AHP peak amplitude, AHP time to peak, and evoked AP latency, accompanying a decrease in the spontaneous AP firing frequency, evoked AP firing frequency, membrane input resistance, membrane time constant, and membrane capacitance of RA projection neurons in adult male zebra finches. These results indicated that carbachol reduces the excitability of RA projection neurons.

Previous studies have shown that the changes of AHP are critical in the modulation neuron activity [23, 24], and AHP regulates firing pattern, firing frequency, and the excitability of neurons [25, 26]. It has been reported that ACh can modulate AHP in many brain neurons [25, 27]. For example, the cholinergic analogue carbachol can produce a depolarization of membrane potential, a higher firing rate, and a smaller AHP in rat oculomotor nucleus motoneurons [28] and neostriatal neurons [25]. However, our results showed that carbachol produced a hyperpolarization of membrane potential, a lower firing rate, and an increase in the AHP peak amplitude and AHP time to peak in zebra finch RA projection neurons, indicating that carbachol reduced the excitability of RA projection neurons by hyperpolarizing the resting membrane potential and changing AP's firing rate and firing pattern, which may be due to the regulation of AHP. In addition, the membrane intrinsic properties also changed during carbachol application. A significant decrease in the membrane input resistance, membrane time constant, and membrane capacitance of RA projection neurons was observed in the present study. The membrane time constant is directly proportional to the product of membrane input resistance and membrane capacitance [29]. The previous study by Hsu et al. showed that a depolarization of membrane potential is concomitant with an increase in the membrane input resistance and firing rate of rat neostriatal neurons [30]. These previous results indicated that the membrane intrinsic properties like the membrane input resistance also correlate with the neuron activity through membrane potential and/or neuron's firing rate. Interestingly, our present results revealed that carbachol application not only induced the depression effect of RA projection neurons through membrane hyperpolarization but also induced the changes of the neuron's membrane intrinsic properties (the decrease in the membrane input resistance, membrane time constant, and membrane capacitance). Taken together, these results indicate that carbachol reduces the excitability of RA projection neurons by hyperpolarizing the resting membrane potential and increasing the AHP and membrane conductance.

The role of ACh has been demonstrated to be complex in different animal species brain and different regions of the brain, and its role is either excitatory or inhibitory [31, 32]. McCormick and Prince found that the ACh-induced hyperpolarization, associated with an increase in the membrane conductance, is prevalent in guinea pig but not cat or rat dorsal lateral geniculate nuclei [31]. Their results showed that the ACh-induced hyperpolarization was mediated by direct activation of postsynaptic muscarinic ACh receptors (mAChRs), which is related to an increase in K^+^ conductance [31]. Ye et al. identified three types of responses to carbachol in rat parafascicular neurons: inhibitory, excitatory, and fast inhibitory followed by slow excitatory, which were mediated by direct activation of postsynaptic mAChRs [32]. The previous study at the single neuron level employing intracellular recording by Salgado-Commissariat et al. [17] demonstrated that the nAChR agonist nicotine produced an increase in frequency of evoked and spontaneous APs and a depression of AHP in adult male zebra finch RA neurons. In contrast, our results of whole-cell patch recordings on adult male zebra finch RA projection neurons showed that the nonselective cholinergic receptor agonist carbachol produced a decrease in the frequency of evoked and spontaneous APs and an increase of AHP. The results from the two studies suggest that the carbachol-induced decrease in the excitability of RA projection neurons may be mediated by mAChRs in RA. It is assumed that a strong inhibitory effect mediated by mAChRs may mask the excitatory effect mediated by nAChRs. We thus hypothesized that ACh may, respectively, act on nAChRs and mAChRs, to increase or decrease the excitability of RA projection neurons in physiological conditions. Further study is required to clarify these differences.

RA projection neurons project to midbrain and brainstem vocal and respiratory structures and send recurrent axon collaterals to both RA neuron types [33, 34]. RA projection neurons also receive excitatory glutamatergic input from HVC and LMAN and receive inhibitory GABAergic input from local inhibitory network formed by RA interneurons [33]. It was reported that RA is an important target nucleus for the modulation of numerous neurotransmitters, including norepinephrine [33, 35], GABA [33], and dopamine [36, 37], and RA also provides a large number of regulatory sites for these neurotransmitters [33]. Individual RA neurons exhibit bursts of activity during singing [34]. The activity of RA projection neurons generates precise neural signals to drive vocal and respiratory motoneurons [19]. Thus, song production is determined by the activity of RA projection neurons. Intrinsic membrane properties play a major role in the integration of synaptic and intrinsic inputs and thereby in the regulation of neuronal behavior [38]. Consequently, based on our experimental results that carbachol can modulate the activity of RA projection neurons by affecting intrinsic membrane properties, we suggest that, under physiological conditions, ACh may play an important role in song production.

## 5. Conclusions

The present study demonstrates that carbachol can reduce the activity of adult male zebra finch RA projection neurons by producing a hyperpolarization of membrane potential and an increase in the AHP and membrane conductance. The results suggest that cholinergic modulation of the songbird premotor nucleus RA may play an important role in song production.

## Figures and Tables

**Table 1:** Measurements of intrinsic properties of RA projection neurons before and during 30 *μ*M carbachol application, as well as after carbachol washout.

Parameters	Pre	Carbachol	*t* values, *p* values	Wash
Membrane potential (mV, *n* = 19)	−59.22 ± 1.25	−66.52 ± 1.03^*∗∗*^	*t* = 6.890, *p* = 1.918*E* − 6	−59.91 ± 1.76
AP threshold (mV, *n* = 19)	−41.02 ± 1.47	−40.48 ± 2.11	*t* = −0.428, *p* = 0.674	−35.20 ± 5.14
Peak amplitude (mV, *n* = 19)	58.14 ± 3.14	57.60 ± 4.26	*t* = 0.246, *p* = 0.808	59.51 ± 4.10
Half-width (ms, *n* = 19)	1.96 ± 0.10	1.84 ± 0.08	*t* = 1.860, *p* = 0.079	2.28 ± 0.32
AHP peak amplitude (mV, *n* = 19)	−23.39 ± 1.02	−27.69 ± 2.02^*∗*^	*t* = 2.689, *p* = 0.015	−22.22 ± 1.84
AHP time to peak (ms, *n* = 19)	37.73 ± 3.64	65.21 ± 12.81^*∗*^	*t* = −2.428, *p* = 0.026	36.98 ± 4.27
Membrane input resistance (MΩ, *n* = 34)	296.18 ± 11.36	215.79 ± 8.85^*∗∗*^	*t* = 10.731, *p* = 2.685*E* − 12	297.45 ± 16.63
Membrane time constant (ms, *n* = 34)	27.68 ± 1.58	15.66 ± 0.76^*∗∗*^	*t* = 9.951, *p* = 1.830*E* − 11	22.31 ± 1.32
Membrane capacitance (pF, *n* = 34)	93.82 ± 4.68	74.76 ± 3.89^*∗∗*^	*t* = 6.848, *p* = 8.141*E* − 8	77.58 ± 4.31

*∗* means *p* < 0.05, *∗∗* means *p* < 0.01, paired *t* test.
